# Does total testicular volume predict testicular volume difference in adolescent males with varicocele?

**DOI:** 10.1590/S1677-5538.IBJU.2017.0652

**Published:** 2018

**Authors:** Ari P. Bernstein, Ethan B. Fram, Amanda North, Anthony Casale, Beth A. Drzewiecki

**Affiliations:** 1 Albert Einstein College of Medicine, NY, USA; 2 Montefiore Medical Center, NY, USA

**Keywords:** Varicocele, Adolescent, Infertility

## Abstract

**Introduction:**

We evaluated the relationship between total testicular volume (TTV) and testicular volume differential (TVD) in adolescent males with varicocele. Both low TTV and high TVD have been independently associated with higher incidences of infertility later in life, but a predictive relationship between TTV and TVD directly has yet to be described.

**Materials and Methods:**

We retrospectively analyzed a database of Tanner 5 boys ages 16-21 who presented with varicocele at a single institution between 2009 and 2017. All patients had a scrotal sonogram prior to surgical intervention. TTV and TVD were calculated for each individual and four non-exclusive groupings of patients were created for statistical analysis. We chose 30 cc as a cut off value for low TTV based on prior studies.

**Results:**

209 patients met our inclusion criteria. Mean age was 18.3 years (16–21, SD 1.7) with a mean total testicular volume of 36 cc (13.5-78.2, SD 11.1). Cut off points of TVD of 20% and TTV of 30 cc were used to separate patients. There were 65 boys (31%) with TTV < 30 cc and 58 boys (28%) with TVD ≥ 20%. Among males with TTV < 30 cc, 23 (35%) had a TVD ≥ 20%. Among males with TTV ≥ 30 cc, 35 (24%) had a TVD ≥ 20%. The relationship between TVD and TTV was found to be non-significant (p > 0.05).

**Discussion:**

Adolescent varicoceles continue to pose a challenge to pediatric urologists. The dilemma of over-aggressive treatment has proven difficult to balance with the risk of infertility. We hoped that elucidating the relationship between TTV and TVD could be useful in identifying patients who are at greater risk for infertility while decreasing the need for more intrusive testing, such as semen analysis, in an adolescent population. We looked at the direct relationship between low TTV and high TVD. In our population, there was a non-significant relationship between TTV < 30 cc and TVD ≥ 20% (p > 0.05) indicating that in adolescents with varicocele, TTV and TVD are independent variables. Our study limitations include the inherent user dependent bias of ultrasound measurements and data collection at a single institution with high ethnic diversity, possibly not comparable to all patient populations.

**Conclusions:**

Low TTV (< 30 cc) itself is not predictive of high TVD (≥ 20%) in adolescent boys with varicocele, despite their reported independent associations with impaired fertility in other studies.

## INTRODUCTION

Varicoceles are defined as abnormal dilation of the pampiniform venous plexus, and are present in 8-16% of adolescent boys (1). Varicoceles are present on the left side more commonly than on the right, which is thought to be secondary to the increased resistance to drainage at the insertion of the left gonadal vein. This resistance to drainage drives the formation of a varicocele and contributes to the sequelae associated with them (2). Although commonly asymptomatic at diagnosis, pediatric varicocele poses a risk for future infertility. Several theories exist to support the functional changes associated with the presentation of varicoceles. One theory suggests that retrograde venous blood flow is associated with increased heat production in the testes and subsequent damage to the developing sperm and Leydig cells (3). Newer theories suggest that the retrograde blood flow increases wall pressure in the veins and contributes to the release of reactive oxygen species that induce damage through oxidative stress (4, 5). Measurement of seminal oxidative stress is possible and has been supported in men presenting with fertility concerns (6). Nevertheless, the exact pathophysiology capable of explaining the great clinical diversity associated with varicoceles remains to be elucidated.

Approximately 15-20% of adolescent boys with varicoceles will discover fertility problems upon entering adulthood (7, 8). Varicocelectomy has been associated in many studies with improved fertility among infertile men with varicocele, as well as with significant increase in volume of the hypotrophic testicle in about 83.8% of adolescent patients who underwent the procedure (9–11). Surgical intervention is thus a reasonable option for pediatric varicoceles that might be at greater risk for causing future fertility issues. Prevention of non-recoverable fertility is the ultimate goal in adolescent varicocele treatment and the current understanding of whom this will be a problem for is unclear.

Differentiating patients for whom repair of a varicocele might improve fertility has been an active area of research and discussion. Numerous strategies have been proposed, including: a varicocele grading scale, testicular asymmetry, semen parameters, hemodynamic studies with Doppler ultrasound, and total testicular volume (TTV) (7). Semen analysis is the most accurate and informative study that can be performed to contribute to a clinical diagnosis of infertility; however, this can only be assessed in sexually mature adolescents. Obtaining a semen analysis can be met with resistance from the family, patient, or both.

Both a low TTV and testicular volume difference (TVD) ≥ 20% have been used to predict potential risk for future problems with fertility in adolescents presenting with varicocele (12, 13). The relationship with lower total motile sperm count (TMC), a commonly used measurement in the clinical assessment of fertility, has been described with both low TTV and a TVD ≥ 20%, but the predictive relationship between TTV and TVD directly has not been described. Using 30 cc as a cut off value for low TTV, we hypothesized that a TTV < 30 cc in adolescents presenting with varicocele will prove to be a statistically significant predictor of TVD ≥ 20%.

## MATERIALS AND METHODS

All patients were identified and demographic information was collected using Clinical Looking Glass (CLG), a database query program developed at our institution. Males aged 16-21 years old and considered to be Tanner V, diagnosed with a varicocele between January 2009 and January 2017, and with completed scrotal sonograms, were included in this study. No patient in this study had a varicocelectomy prior to the sonogram. Left and right testicular volumes were calculated using the Lambert equation: Testicular volume = (Length)×(Width)×(Height)×(0.71) (14). TTV was calculated by taking the sum of the left and right testicular volumes. Additionally, TVD was calculated using the following formula: (Volumeright - Volumeleft)/Volumeright×100 (12).

Four non-exclusive groupings of patients were used in statistical analysis: 1) TTV ≥ 30 cc with TVD ≥ 20%, 2) TTV ≥ 30cc with TVD < 20%, 3) TTV < 30 cc with TVD ≥ 20%, and 4) TTV < 30 cc with TVD < 20%. Considering that ipsilateral hypotrophy has been strongly associated with varicocele, we included boys with TVD ≥ 20% into the appropriate groups only if the asymmetry was concordant with reported varicocele laterality (15). Patients with bilateral varicoceles were included and evaluated for left-sided hypotrophy in the setting of TTV as well. Chi-square tests were performed using IBM's SPSS version 20.

## RESULTS

Two hundred and nine patients met our inclusion criteria. Basic demographics and testicular volumes are shown in Table-1. The majority of patients had either a left-sided (66.0%) or bilateral (30.6%) varicoceles, with 3.4% having a right-sided varicocele. Patients were then divided by testicular volume differential and total testicular volume (Table-2).

**Table 1:** Baseline demographic characteristics of patients who met the inclusion criteria.

Characteristics	Value
Total no. patients	209
Mean age ± SD at presentation (in years)	18.3 ± 1.7
	White = 38 (18%)
	Black = 55 (26%)
Race (%)	Other = 77 (37%)
	Needs clarification = 6 (3%)
	Decline = 32 (15%)
	Multiple races = 1 (0.5%)
Left varicocele (%)	138 (66.0%)
Right varicocele (%)	7 (3.4%)
Bilateral varicoceles (%)	64 (30.6%)
Mean R testicular volume (cc) ± SD	18.9 ± 6.2
Mean L testicular volume (cc) ± SD	17.1 ± 6.0
Mean TTV (cc) ± SD	36.0 ± 11.1

**Table 2:** No. patients organized by TVD and TTV.

	TVD ≥ 20%	TVD < 20%
TTV < 30 cc	23 (11%)	42 (20%)
TTV ≥ 30 cc	35 (17%)	109 (52%)

Mean age ± SD at presentation was 18.3 ± 1.7. Mean right testicular volume ± SD was 18.9 ± 6.2 cc, while mean left testicular volume ± SD was 17.1 ± 6.0 cc. Mean TTV ± SD was 36.0 ± 11.1 cc. 65 boys (31%) presented with TTV < 30 cc while 144 (69%) presented with TTV ≥ 30 cc. 58 boys (28%) presented with TVD ≥ 20% while 151 (72%) presented with TVD < 20%.

**Figure 1:**
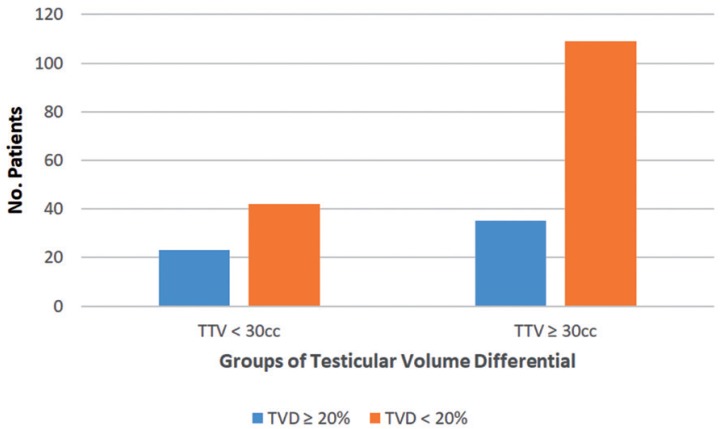
Number of males with TVD ≥ 20% and < 20% grouped by TTV.

The number of adolescent males with and without TVD ≥ 20% for the groups with TTV < 30 cc and TTV > 30 cc respectively are presented in Figure-1. Among the males with TTV < 30 cc, 23 (35%) had a TVD ≥ 20% while 42 (65%) had a TVD < 20%. Among the males with TTV ≥ 30 cc, 35 (24%) had a TVD ≥ 20% while 109 (76%) had a TVD < 20%.

The relationship between testicular volume difference ≥ 20% and total testicular volume at a threshold of 30 cc was found to be non-significant (p = 0.0978). This analysis was repeated in our subset of unilateral varicocele adolescents, excluding those with bilateral varicoceles. The result of this chi-square was also found to be non-significant (p = 0.3373).

## DISCUSSION

Adolescent varicoceles continue to pose a challenge to pediatric urologists. While there is a high prevalence in the general population, estimated at 8-16%, only about 1 / 5th of these cases will result in fertility problems later in life (11). We have limited methods to predict whether an adolescent varicocele will ultimately impact fertility. Both TTV and TVD are useful parameters but they appear to be independent in their relationship to semen analysis (7, 12).

In a landmark study by Diamond et al., a strong relationship between total motile sperm count (TMC) and TVD was found in a group of Tanner 5 boys with left-sided varicoceles. Greater than 20% asymmetry was associated with the greatest reduction in TMC, with TMC findings approaching the normal range as TVD decreased (12). This study suggests that TVD of greater than 10% could be considered a threshold for concern and supported the use of 20% TVD as an indication for surgery (7). This study also argues that total testicular volume is a potentially reliable marker to assess fertility potential, in the finding that TTV < 30 cc more than quadrupled the odds of having a clinically low TMC (12). In another study by Diamond et al., it was found that the association of low TTV (36 cc as a cut off value) and low TMC was also proportional to TVD, so that a patient with a high asymmetry in testicular size was more likely to have a lower TMC if they also had a low TTV (13). Further analysis on how this patient population compared to ours is not available from this abstract.

In adults, semen analysis is the most objective tool to evaluate male fertility potential. In the adolescent population it has been less widely accepted, given the social and practical challenges to obtaining the specimen. In a study by Fine et al., it was found that 53% of pediatric urologists out of a sample of 168 providers felt uncomfortable asking patients for a semen analysis, with 90% of them claiming to never order such an analysis (16). Semen analysis in this population can be difficult to obtain and often controversial in its collection, as is evidenced by the lack of many studies using semen analysis in these young populations. For this reason, multiple surrogate markers of fertility potential have been investigated. Varicocele grade has demonstrated to be an unreliable determinant of future asymmetry, whereas hemodynamic studies using Doppler ultrasound have been found to be helpful determinants when used in conjunction with testicular asymmetry by measuring peak retrograde flow (PRF). Three distinct studies discovered that a combination of 20% asymmetry or greater and a PRF greater than 38 cm / s to be strongly linked with worsening asymmetry over time (11, 17–19). Increased TVD and decreased TTV have been associated with abnormally low TMC values, suggesting their relationship to infertility later in life (12). Both of these measurements are easier to obtain relative to other surrogate markers of fertility potential with outpatient ultrasonography, and, if shown to be repeatedly significant predictors of clinically-diagnosed infertility later in life, could potentially negate the need to obtain semen samples in adolescents.

In our study, we evaluated whether low TTV itself was predictive of high TVD. If low TTV and high TVD are independently related to low TMC, we hoped to find a more meaningful relationship between these two factors that could be used in absence of semen analysis. We believed that elucidating this relationship could be important in preventing boys from needing to provide a semen sample which is somewhat controversial and often difficult to obtain in this younger patient population. In our population of 209 boys presenting with varicocele, there was a non-significant relationship between TTV < 30 cc and TVD ≥ 20% (p > 0.05). Considering that inclusion of bilateral varicocele cases could have perhaps masked a potential effect of unilateral varicoceles on asymmetry, an additional chi-square analysis was performed on the subset of unilateral varicoceles, excluding those with bilateral varicoceles. This analysis was also found to be non-significant (p > 0.05), supporting our study's findings.

Even though the results were not statistically significant, among the patients with TTV < 30 cc there was a higher percentage with TVD ≥ 20% relative to the patients with TTV ≥ 30 cc. It is possible that TTV < 30 cc is indicative of a more global effect of the varicocele on the testes rather than only on one side. Specifically, a varicocele affecting the testicles significantly enough might cause both testes to suffer rather than just one. This mechanism would render the TTV lower and make the difference in volume between the two less of a factor, which would support our non-significant finding. It may also represent a patient population whose testicular size may be diminished for other reasons, such as syndromes or drug use. We did not evaluate these other comorbid conditions in this study. We felt that boys with TTV < 30 cc would have a higher incidence of asymmetry since both were independently found to be indicative of lower TMC and were perhaps more connected than previously understood. While TVD and TTV independently impart a worse prognosis on semen analysis parameters, it is unknown which comes first and how the two relate to each other. Our findings indicate that TTV and TVD may not be directly related, and the question of whether or not one marker or the other is a better surrogate of fertility potential remains unanswered.

One study by Diamond et al. used a threshold volume of 36 cc in their description of low TTV when compared with associated TMC values (13). We found non-significant results with that volume threshold as well, and chose to keep with our usage of 30 cc which is more commonly described in the literature.

Our study has limitations that should be known. One inherent limitation is the retrospective nature of this study. Additionally, ultrasound usage as a determinant of testicular volume measurements is a user dependent tool. Even slight errors in one or more of the 3 testicular dimensions can result in significantly different testicular volumes, and therefore must be considered a possible source of error (20). Both Prader and Rochester orchidometers have been shown to be insensitive relative to sonograms in terms of accurately measuring smaller testicular volumes and especially testicular volume differentials (21). For this reason, our testicular volumes and differentials were calculated using sonograms alone. Additionally, our population was gathered at a single center in an area with high ethnic diversity and may not be comparable to all patient populations. Larger studies should be performed that include analyses of subgroups to see if diversity masks predictive value among certain ethnic subtypes. Lastly, our study focuses on TTV and TVD, and does not include semen analyses on our patients to further corroborate our results.

## CONCLUSION

Low TTV (< 30 cc) itself is not predictive of high TVD (≥ 20%) in adolescent boys with varicocele despite their reported independent associations with impaired fertility in other studies. Further research must be conducted to uncover more practical and valuable surrogate markers of fertility potential that will help physicians identify boys at greater risk of developing infertility for early intervention purposes.
